# Does Genetic Background Affect Susceptibility to Alcohol-Induced Heart Disease?

**DOI:** 10.1016/j.jacadv.2025.102049

**Published:** 2025-08-08

**Authors:** Samuel J. Tu, Emma M. Rath, Eleni Giannoulatou, Diane Fatkin, Christopher X. Wong

**Affiliations:** a Centre for Heart Rhythm Disorders, University of Adelaide, Adelaide, Australia; b Flinders Medical Centre, Bedford Park, Adelaide, Australia; c Victor Chang Cardiac Research Institute, Darlinghurst, New South Wales, Australia; d School of Biomedical Sciences, UNSW Sydney, Kensington, New South Wales, Australia; e School of Clinical Medicine, UNSW Sydney, Kensington, New South Wales, Australia; f Cardiology Department, St Vincent’s Hospital, Darlinghurst, New South Wales, Australia; g Department of Cardiology, Royal Adelaide Hospital, Adelaide, Australia

**Keywords:** alcohol, dilated cardiomyopathy, genetic, polygenic risk

**Figure:**
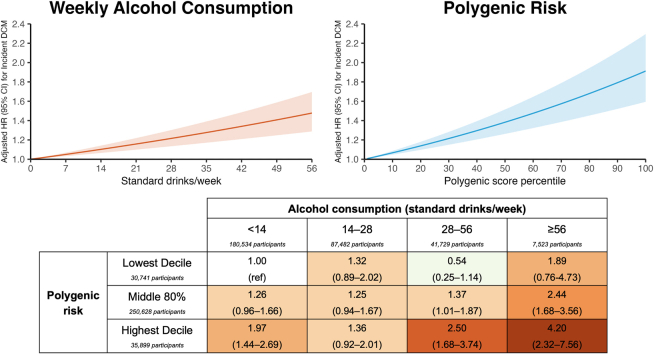

Dilated cardiomyopathy (DCM) is a heterogeneous condition that can have genetic and environmental etiologies. Habitually high levels of alcohol consumption may result in a specific DCM phenotype known as alcohol-induced cardiomyopathy. However, whether lower levels of alcohol intake have adverse or even beneficial effects on cardiac function is unclear.^1^^,^^2^ Moreover, whether individual profiles of background genetic variation influence susceptibility to alcohol-induced cardiomyopathy is unknown.

**What is the clinical question being addressed?**

Does genetic predisposition affect the risk of dilated cardiomyopathy associated with alcohol consumption?

**What is the main finding?**

An additive risk of alcohol consumption exists among individuals with a genetic predisposition to dilated cardiomyopathy.

We analyzed a prospective cohort study of over 500,000 individuals enrolled in the UK Biobank (North-West Multi-centre Research Ethics Committee, #94505). We excluded participants who had a prior diagnosis of any cardiomyopathy, former/never alcohol consumers, current alcohol consumers without recorded consumption, and participants of non-European ancestry. Alcohol consumption was evaluated as UK standard drinks (8 g alcohol) per week. Polygenic risk scores (PRSs) were calculated in all 317,268 participants from whole genome sequencing data using the PGS000666 PRS (referred to ongoing as DCM-PRS).^3^ The DCM-PRS is a 28-single nucleotide variant score based on indexed left ventricular end-systolic volume that is reported to correlate strongly with incident DCM.^3^ Participants of non-European ancestry were excluded in this analysis because the DCM-PRS used was developed using a cohort that is genetically of European ancestry (98.2%) and validated in a cohort of predominantly European ancestry. The distribution of DCM-PRS scores within the cohort was ranked and expressed as mean scores and percentiles. Incident DCM was ascertained from the first occurrence of International Classification of Diseases, 10th Revision codes I42.0, I42.6, I42.8, or I42.9. Stratified Cox proportional hazard models were used to assess continuous and categorical associations of alcohol consumption and polygenic risk with incident DCM. Baseline hazards were stratified by sex to meet the proportional hazards assumption, and models were adjusted for age, body mass index, smoking, self-reported exercise, and baseline comorbidities including hypertension, diabetes, hyperlipidemia, valvular disease, and ischemic heart disease as time-varying covariates. Interactions between alcohol consumption and polygenic risk were assessed with and without the inclusion of a multiplicative interaction term.

Among 317,268 participants, 1,423 incident DCM events occurred over a median follow-up of 13.6 years (4,193,884 person-years). Those who developed DCM were older (median [IQR]: 61 [56, 65] vs 58 [51, 63] years) and more likely to have ischemic heart disease (12.9% vs 4.7%), valvular heart disease (2.2% vs 0.7%), hypertension (40.4% vs 26.0%), and diabetes (8.1% vs 4.0%; *P* < 0.001 all).

Subjects who developed DCM consumed more alcohol (13.2 vs 12.0 standard drinks/week) than those who did not develop DCM (*P* < 0.001). For every 7-standard drink (56 g alcohol)/week increase in alcohol consumption, there was a 5% increase in the hazard for incident DCM (HR: 1.05; 95% CI: 1.03-1.07; *P* < 0.001). There was no significant improvement in goodness of fit when alcohol was analyzed in a nonlinear model as a restricted cubic spline (*P* = 0.10).

Mean DCM-PRS percentiles were greater in those with incident DCM (56.3% vs 50.9%, *P* < 0.001). For every 10-percentile DCM-PRS increase, there was a 7% increase in incident DCM (HR: 1.07; 95% CI: 1.05-1.09; *P* < 0.001). Compared to the lowest decile of DCM-PRS, the middle 80% of participants was associated with a 23% higher (HR: 1.23, 95% CI: 1.01-1.52) and the highest decile 48% higher (HR: 1.48, 95% CI: 1.28-1.70) hazard of DCM.

There was an increased, additive risk of incident DCM associated with both increasing alcohol consumption and DCM-PRS (Figure 1, top panel). While a possible multiplicative interaction was suggested when alcohol consumption and DCM-PRS were analyzed categorically (*P* = 0.02) (Figure 1, bottom panel), no significant interaction was observed when they were modeled as continuous variables (*P* = 0.40). Sensitivity analyses restricting incident DCM events to International Classification of Diseases, 10th Revision codes I42.0 and I42.6, excluding those with ischemic heart disease or valvular disease, excluding those whose MRIs were involved with DCM-PRS derivation, or including those who were former/never alcohol drinkers did not materially affect results.

**Figure 1:**
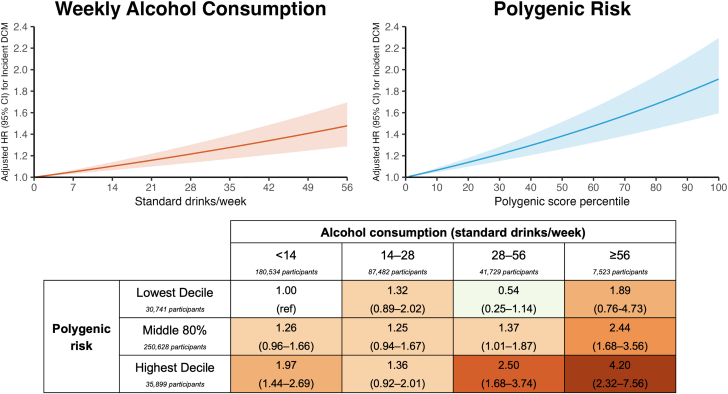
**Associations of Alcohol Consumption, Polygenic Risk, and Incident Dilated Cardiomyopathy** Top panel: Associations of increasing continuous alcohol consumption and polygenic risk score percentile with hazard of incident DCM (left and right, respectively). Shaded areas refer to 95% CIs. Bottom panel: Heat map demonstrating HRs and 95% CIs for incident DCM in specific alcohol and polygenic risk categories. Cox proportional hazard models for continuous (top) and categorical (bottom) associations were stratified by sex to meet the proportional hazards assumption and adjusted for age, body mass index, smoking, self-reported exercise, and baseline comorbidities including hypertension, diabetes, hyperlipidemia, valvular disease, and ischemic heart disease as time-varying covariates. Standard drinks represent the UK definition of 8 g of alcohol. DCM = dilated cardiomyopathy.

Many questions remain regarding the relationship between alcohol and DCM.^1^ Alcohol-induced cardiomyopathy has been documented at extreme levels of consumption, but the incremental risk of DCM with moderate alcohol has not been well characterized. This analysis demonstrates an increasing dose-response relationship between alcohol and DCM across the range of alcohol consumption. Our data suggests that even low or minimal alcohol intake may be associated with increased DCM risk, as with other cardiovascular diseases.^4^ This contrasts with previous longitudinal studies evaluating the broader clinical diagnosis of heart failure that have demonstrated mixed and even protective associations of alcohol at low levels.^2^

Here we also find that alcohol consumption is consistently associated with increased DCM risk across the spectrum of polygenic predisposition. The effects of alcohol and polygenic risk on incident DCM appear to be additive, with no significant multiplicative effect. Our results extend previous observations that pathogenic rare variants in cardiomyopathy-associated genes are relatively enriched in patients with alcohol-induced cardiomyopathy.^5^ Collectively, these findings suggest that alcohol may unmask or accelerate the development of clinical DCM in genetically predisposed individuals.^1^

Strengths of our analysis include the cohort design and specific incident DCM events compared to general clinical heart failure studied previously. Limitations include self-reported alcohol consumption, lack of generalizability to individuals of non-European ancestry, inability to definitively exclude concomitant ischemic heart disease, and restriction to common DCM variants, acknowledging that genetic influences are likely to be extensive and impact other relevant parameters such as alcohol metabolism, comorbidities, and more.

In conclusion, both alcohol and genetic susceptibility were associated with DCM risk. There is a dose-response association of alcohol with incident DCM in general and an additive risk of alcohol consumption among individuals with a genetic predisposition to DCM. Our findings have important implications for alcohol intake recommendations at both a population level and in individuals with elevated genetic risk.

## Funding support and author disclosures

This work is supported by a Medical Research Future Fund (MRFF) Cardiovascular Health Mission Grant (MRF2017687). Dr Fatkin is supported by an NSW Health Senior Cardiovascular Researcher Grant. Dr Wong is supported by an NHMRC Investigator Grant. All other authors have reported that they have no relationships relevant to the contents of this paper to disclose.
